# Clostridium difficile infections in teaching hospital in northern Finland

**DOI:** 10.1186/s12879-018-3663-y

**Published:** 2019-01-11

**Authors:** M. Marttila-Vaara, P. Ylipalosaari, H. Kauma

**Affiliations:** 1 Research unit of Internal Medicine, Oulu University Hospital and University of Oulu, Oulu, Finland; 2 Department of Infection Control, Oulu University Hospital, Box PL 20 FIN-90029 OYS, Oulu, Finland

**Keywords:** Diarrhea, Clostridium difficile, Healthcare associated infection, Epidemiology

## Abstract

**Background:**

The aim of this study to compare the incidence of Clostridium difficile (CD) infections in the five university hospital districts in Finland based on national register. The clinical findings of CD cases in the Oulu University Hospital (OUH) in one-year cohort were also analyzed.

**Methods:**

The numbers of the CD cases from the national register were used for the hospital district comparison. A retrospective cohort study was conducted among all adult (> 16 years) patients treated in the OUH in 2013, who had positive CD toxin B gene test in stools. The selection of the cohort was based on the data from the OUH microbiology laboratory and the clinical characteristics were collected from hospital records.

**Results:**

The incidence of CD findings in 2013 was higher in the OUH district than in the other four university hospital districts: 159 vs. 70 to 84 per 100,000 inhabitants.

In 2013, 261 patients had CD infection treated in the OUH. The yearly number of CD cases treated in the OUH in 2009–2016 varied between 221 and 287, and the corresponding proportion of positive CD findings out of all samples taken varied from 10.0 to 17.8%. A recurrent infection was seen in 58 patients (22%) while the all-cause 30 day mortality was 7.3%.

**Conclusions:**

Diagnostic strategies differed nationally, which may explain the differences in CD incidence between the university hospital districts. In the OUH, no increase in the number of CD infections was seen in 2009–2016. Main characteristics of the patient cohort in the OUH were in harmony with earlier literature.

## Background

Clostridium difficile infection (CDI) is a leading cause of hospital -associated gastrointestinal illness which varies from of illness, which varies from asymptomatic infection, diarrhea, colitis, and pseudo-membranous colitis to death [1]. *C. difficile* expresses two key virulence factors; the exotoxins, toxin A (TcdA) and toxin B (TcdB), which are glucosyltransferases targeting host-cell monomeric GTPases [2]. In addition, some hypervirulent strains produce a third toxin, binary toxin or *C. difficile* transferase (CDT), which may contribute to the pathogenesis of CDI [3]. The innate immune responses to *C. difficile* and its toxins are very important in the pathogenesis of CDI, although the adaptive immune responses have also been reported to influenze the severity of CDI [4]. Diagnostic approaches are complex due to the availability of multiple testing strategies. Multistep algorithms using polymerase chain reaction (PCR) for the toxin gene(s) or single-step PCR on liquid stool samples have the best test performance characteristics [5].

Risk factors for *C. difficile* infection (CDI) have typically been recent antibiotic exposure, advanced age, use of proton pump inhibitor, length of stay in healthcare settings, serious underlying illness, and immunocompromised conditions [6, 7]. Patients with CDI typically have extended lengths of stay in hospitals, and CDI is a frequent cause of large hospital outbreaks. The cost of treatment is expensive and the financial burden on the medical system is substantial [6, 8]. Earlier literature on this topic in Finland has been controversial. It has been suggested that the figures of health care associated CDI have decreased due to better infection control althought diagnostic methods have become more sensitive [9]. In the other study the level of fluoroquinolone use has been associated to regional differences of CDI [10]. We compare the incidence of CDI according to the National Infectious Diseases Register (NIDR, National Institute for Health and Welfare, Helsinki, Finland) in five university hospital districts in Finland. Moreover, we characterize CDI patients treated in the Oulu University Hospital (OUH) in one-year cohort.

## Methods

The study was conducted in the Oulu University Hospital which is a tertiary care teaching hospital covering an area of approximately 407,000 inhabitants, with 985 beds and approx. 260,000 treatment days/year. The incidence of CD findings in five university hospital districts in Finland was compared using the data of the National Infectious Diseases Register (NIDR) of National Institute for Health and Welfare in the year 2013 [11]. Noteworthy is that this register includes not just patients treated in the OUH but also patients treated in non-teaching hospitals, health centers and in private clinics in this district. Therefore the numbers of the cases treated in the OUH are clearly lower than cases in the whole OUH district.

The trend of *C. difficile* figures in the OUH the years 2010–2016 was analyzed according to the CD findings diagnosed in the microbiology laboratory of the OUH. The following data was collected from the hospital database: age, comorbidities, hospitalization history, antibiotic exposure, procedures, mortality data (30 d), CDI recurrences, and length of hospital stays. All cases underwent full medical-record review by an infectious diseases specialist (M. M-V.) to collect information on coexisting medical conditions, medication exposures, first laboratory-confirmed recurrences (i.e. positive specimen within 2 to 8 weeks after the last positive test). The term ‘recurrent CDI’ was used to denote either relapse or reinfection, as the diagnosis and management of both forms of recurrence are similar, and the two mechanisms are rarely differentiated in clinical practice.

The method for CDI detection was a polymerase chain reaction (PCR) -based, nucleic acid amplification test (NAAT) on CDI toxin B gene, *tcdB*, without preceding fecal culture. The samples were analyzed using GenomEra CDX™ test (Abacus Diagnostica, Turku, Finland). The incidences of *C. difficile* in (CD) gene typing CD pathogenicity (PaLoc) and CDT locus virulence factors were tested using in-house multiplex PCR method, using gel electrophoresis to test for *tcdA* and *tcdB* whether there is toxin production and whether the regulatory elements of the genes are mutated, like with deletions (e.g. ribotype 027). [12] All samples with differing gene profiles were sent to the National Institute for Health and Welfare (THL, Turku, Finland) for further ribotyping.

### Statistical analysis

Summary statistic are presented as mean and standard deviation (SD). The χ^2^ test and Fisher exact test was used to compare categorical variables, and the t-test and the Mann-Whitney U-test were used to compare continuous variables. *p* values > 0.05 were considered as statistically significant. The statistical analyses were performed with IBM SPSS Statistics for Windows, version 22 (IBM Corp., Armonk, NY, USA).

## Results

Five university hospitals used different methods for CDI diagnostics (Table 1). In the study hospital (OUH) district the incidence of CD findings per 100,000 inhabitants (based on national register) was higher compared with other university hospital districts (159 vs. 70–84).

**Table 1:** Comparison of the incidence of Clostridium difficile cases per 100,000 inhabitants in five university hospital districts in Finland basing on the national register in the year 2013

Hospital district	Population (million)	Number of CD cases in national register	Incidence per 100,000 inhabitants	Test method
Hospital A	1.6	1173	73	F-Cldi culture and EIA
Hospital B	0.5	368	70	F-CldiAg + F-Cldi culture
Hospital C	0.5	420	84	F-Cldi culture and GDH
Hospital D	0.4	638	159	F -CldTNhO
Hospital E	0.2	168	84	NAAT for tcdB

*F* faecal, *Cldi Clostridium difficile*, *EIA* enzyme immuno assay, *Ag* antigen, *GDH* glutamate dehydrogenase assay, *NAAT* nucleid acid amplification test, *tcdB* toxin B gene, *NhO* nucleid acid assay

In 2013, totally 6948 stool samples for CD toxin determination were examined in the OUH laboratory (Nordlab). From 1015 positive samples (14.5% of all), 261 different patients with 391 positive findings were treated in the study hospital. The main characteristics of the study population are shown in Table 2. The mean age was 65 yrs. (range 16–102), while 37% were older than 75 yrs., 22% were diabetic and 29% immunocompromised (defined as a malignant disease or an immunosuppressive disease or treatment). Chronic inflammatory bowel disease was uncommon. Surprisingly, diarrhea as a symptom the day the stool sample was collected was mentioned in the patient documents in only 58% of cases (no data in 28/261 11% of cases).

**Table 2:** Main characteristics of adult CD patients (*N* = 261) treated in Oulu University Hospital in 2013

Variable	
Gender (M/F)	125/136 (48/52%)
Age (yrs, mean, SD)	64.8 (19.1)
Department^a^ (medicine/surgery)	192/69 (74/26%)
Positive CD culture	32 (12%)
Genotyping	10 (3.8%)
Diabetes	58 (22%)
Immunosuppressive	76 (29%)
IBD^b^	7 (2.7%)
Chronic renal disease	18 (6.9%)
Preceding antibiotics	
Cephalosporins	127 (48%)
Other beta-lactams	102 (39%)
Fluoroquinolones	37 (14%)
Clindamycin	20 (7.7%)
Vancomycin	27 (10%)
Antifungals	10 (3.8%)
Other antibiotics	19 (7.3%)
No data	34 (13%)

^a^The specialty where the patient was treated. In surgery gynaecology, otorhinolaryngology and ophthalmology are included. ^b^Chronic inflammatory bowel disease. *CD* Clostridium difficile

The treatment for the first episode of CDI was as follows: metronidazole in 167 cases (64%), per oral vancomycin in 42 (16%) and metronidazole and vancomycin combination therapy in 10 (3.9%). Fidaxomicin was given in four cases (1.6%). In 22 cases (8.4%) the only treatment was the cessation of the ongoing antibiotics and in 16 (6.1%) no data was found. In two situations, fecal transplantation was performed and in one case of toxic megacolon colectomy was performed. Recurrent infection was seen in 58 patients (22%). The number of recurrences was one in 34 patients, two in 14, three in six, four in two and five recurrences in two cases. There was no difference in the risk of recurrent infection between those treated with metronidazole or vancomycin (35/167, 27% vs. 13/42, 31% *p* = 0.17).

The all-cause 30 day mortality rate of the patients was 7.3% The majority of the cases (74%) were identified in the department of medicine, while the mortality rate was more than two times higher among surgical cases (13.0% vs. 5.2%, *p* = 0.03). The number of recurrences was significantly higher among survived CD patients (*p* = 0.003).

Ribotype analysis was performed in only 17 cases. Ribotype 178 was the most common, seven cases following 023 (four cases), 126 (two cases), and 014, 035, 045 and 143 (each one case). No ribotype 027 cases were identified.

As Table 3 shows, there was no increase either in the CD cases treated in the OUH in 2009–2016 or in the proportion of positive CD findings per all studied samples.

**Table 3:** Number of Clostridium difficile patients treated in the Oulu University Hospital in 2009–2016

Year	Number of patients with positive samples	Number of patients with negative samples	Number of all patients with samples taken	The percentage of positive samples of all samples
2009	239	1103	1342	17.8
2010	221	1190	1411	15.7
2011	263	2376	2639	10.0
2012	231	1313	1544	15.0
2013	261	1547	1808	14.4
2014	287	1551	1838	15.6
2015	267	1507	1774	15.1
2016	230	1586	1816	12.7

Also the yearly number of all and negative faecal samples and proportion of positive samples are shown

## Discussion

Our results show that the incidence of CD cases varied in five university hospital districts probably due the different diagnostic methods. Based on data from the OUH laboratory, the yearly numbers of CD cases treated in the OUH were quite constant during the years 2009–2016. In our one-year cohort, the number of recurrence was 22% and the 30 day all-cause mortality rate 7.3%. It is also noteworthy that the mortality among the patients treated in surgical wards was significantly higher than among the patients treated in medical wards. Because of the figures isallcause mortality we cannot exclude the contribution of the underlying diseases.

There may be several explanations why our hospital district had highest incidence of CD infections. We have used PCR-based diagnostics since the autumn 2010 whereas three other university hospitals used culture method when the survey was performed. In a population-based surveillance, the adoption of NAAT instead of toxin enzyme assay for diagnosis of CDI showed increase of 43–67% in CDI incidence [12]. In a European review, the mean sensitivity of the PCR based methods was 86% and the mean specificity 97%, both much higher than with other methods [13]. According to the current guidelines two or three steps algorithm should be use for diagnosing CDI instead of stand-alone test for toxin gene detection by NAAT or PCR [14, 15]. It is possible that some of the cases detected by NAAT or PCR represent colonization rather than true infection, given that NAAT detects the presence of the organism but not necessarily, if it is disease-causing [16]. The rate of asymptomatic colonization in non-hospitalized adults is estimated to be 2% and up to 26%, in those with health care exposures [17, 18]. Asymptomatic colonization may explain some of our CD findings, because only 58% cases had diarrhea marked as a previous symptom, leaving a doubt that even almost 40% of the samples had no indication. Actually, this is against the current guidelines, which support to test only unformed stool samples [14, 15, 19]. One-step procedure performed by a highly sensitive method demands appropriate sample collection. Unless adjustments based on methodology and population prevalence are considered, hospitals participating in public reporting of CDI rates will be at a disadvantage for performing the most sensitive testing methodologies [1, 16]. So, the methodological differences between regional laboratories hamper the comparisons between the hospital districts. One reason for differences in five university hospitals may also due to problems with the data collection from the hospital districts to the national CDI register.

Overall use of antibiotic agents is associated with a 3-fold increased risk of community-acquired CDI, but it has also been detected substantial variation in risk associated with different antimicrobial classes, with fluoroquinolones and clindamycin associated with the greatest enhancement of risk [20]. In our study showed that most often cephalosporins (49%), other β-lactams (45%) and fluoroquinolones (14%) were used before CDI. In our material 7.7% were using clindamycin previously, even though it has been previously described as a high-risk antibiotic [20]. That cephalosporins were used most often preceding CDI reflects our guidelines, where cefuroxime, a second-generation cephalosporin, is the first line antibiotic in several diagnoses, like community acquired pneumonia, pyelonephritis and community acquired bacteremia.

In our study mean recurrence rate was 22%. Recurrence rates for health care–associated CDI have been reported to vary from 5 to 50%, with an average of 20% [21, 22]. Also higher recurrence rates have been variously reported, from 3% to even 65% [23, 24]. There are three risk factors, which predict the risk for recurrent CDI: age > 65 years, severe underlying disease and continued use of antibiotics for non-CDI infections [25]. In our study mean age was 64.8 years, at least 61% had a significant comorbidity and 85% had a continued use of antibiotics for non-CDI infections.

Cochrane review in 2013 states on CDI treatment that current evidence leads to uncertainty whether mild CDI needs to be treated [26]. The studies provide little evidence for antibiotic treatment of severe CDI as many studies excluded these patients. Improvement of the patient’s clinical condition and prevention of spread of *C. difficile* infection to the other patients should be the two goals of therapy. Therefore an antibiotic that brings both symptomatic cure and bacteriologic cure [25] is to be chosen. In our study, the treatment options mimicked the existing guidelines, 64% were treated with metronidazole, 16% with peroral vancomycin, metronidazole and vancomycin combination therapy was used in 3.9% of cases. Fidaxomicin was used only in four cases, but it has been reported that fidaxomicin provides improved sustained cure rates in patients with CDI compared with vancomycin [27]. There were no statistical differences in treatment options between survivors and fatal cases in 30-day all case mortality. Two patients had fecal microbiota transplantation that year, since then its use has widened significantly in our university hospital district.

Our analyses have limitations. Collecting data retrospectively always has its limitations in the form of missing data. In addition, the case definition relied solely on positive results on *C. difficile* toxin or molecular assay because diarrhea is usually poorly documented in charts and existing guidelines for laboratory practice recommend *C. difficile* testing only on unformed stools. Laboratories should adopt stricter policies to reject formed stools when transitioning to NAAT.

## Conclusions

Our PCR-based approach gave higher incidence of CD infections than other four university hospital districts in Finland. However, the number of the cases treated in the OUH remained quite stable and the main clinical characteristics of the cohort were in harmony with earlier literature. The quality of stool samples needs more critical evaluation in our hospital because almost 40% of the samples were formed stools, i.e. they actually did not represent diarrhea and might have therefore no indication for CD analysis.

## Abbreviations

A (TcdA): Toxin A
B (TcdB): Toxin B
CDI: Clostridium difficile infection
CDT: Clostridium difficile transferase
GTPases: Glucosyltranspherases
NAAT: Nucleic acid amplification test
NIDR: the National Infectious Diseases Register
OUH: Oulu University Hospital
PCR: Polymerase chain reaction

## References

[1] Leffler DA, Lamont JT (2015). Clostridium difficile infection. N Engl J Med.

[2] Kuehne SA, Cartman ST, Heap JT, Kelly MT, Cockayne A, Minton NP (2010). The role of toxin a and toxin B in Clostridium difficile infection. Nature.

[3] Rupnik M, Grabnar M, Geric B (2003). Binary toxin producing Clostridium difficile strains. Anaerobe.

[4] Sun X, Hirota SA (2015). The roles of host and pathogen factors and the innate immune response in the pathogenesis of Clostridium difficile infection. Mol Immunol.

[5] Martínez-Meléndez A, Camacho-Ortiz A, Morfin-Otero R, Maldonado-Garza HJ, Villarreal-Treviño L, Garza-González E (2017). Current knowledge on the laboratory diagnosis of Clostridium difficile infection. World J Gastroenterol.

[6] Evans CT, Safdar N (2015). Current Trends in the Epidemiology and Outcomes of Clostridium difficile Infection. Clin Infect Dis.

[7] Bignardi GE (1998). Risk factors for Clostridium difficile infection. J Hosp Infect.

[8] Desai K, Gupta SB, Dubberke ER, Prabhu VS, Browne C, Mast TC (2016). Epidemiological and economic burden of Clostridium difficile in the United States: estimates from a modeling approach. BMC Infect Dis.

[9] Mentula S, Kotila SM, Lyytikäinen O, Ibrahem S, Ollgren S, Virolainen A (2017). Clostridium difficile infections in Finland, 2008-2015: trends, diagnostics and ribotypes. Eur J Clin Microbiol Infect Dis.

[10] Kanerva M, Ollgren J, Voipio T, Mentula S, Lyytikäinen O (2015). Regional differences in Clostridium difficile infections in relation to fluoroquinolone and proton pump inhibitor use, Finland, 2008-2011. J Infect Dis.

[11] https://thl.fi/fi/tilastot-ja-data. National Institute for Health and Welfare, Helsinki, Finland.

[12] Gould CV, Edwards JR, Cohen J, Bamberg WM, Clark LA, Farley MM, Johnston H, Nadle J, Winston L, Gerding DN, McDonald LC, Lessa FC (2013). Effect of Nucleid acid amplification testing on population-based incidence rates of Clostridium difficile infection. Clin Infect Dis.

[13] Crobach MJ, Dekkers OM, Wilcox MH, Kuijper EJ (2009). European society of clinical microbiology and infectious diseases (ESCMID): data review and recommendations for diagnosing Clostridium difficile-infection (CDI). Clin Microbiol Infect.

[14] Crobach MJT, Planche T, Eckert C, Barbut F, Terveer EM, Dekkers OM, Wilcox MH, Kuijper EJ (2016). European Society of Clinical Microbiology and Infectious Diseases: update of the diagnostic guidance document for Clostridium difficile infection. Clin Microbiol Infect.

[15] McDonald LC, Gerding DN, Johnson S, Bakken JS, Carroll KC, Coffin SE, Dubberke ER, Garey KW, Gould CV, Kelly C, Loo V, Sammons JS, Sandora TJ, Wilcox MH (2018). Clinical practice guidelines for Clostridium difficile infection in adults and children: 2017 update by the Infectious Diseases Society of America (IDSA) and Society for Healthcare Epidemiology of America (SHEA). Clin Infect Dis.

[16] Nagy E (2018). What do we know about the diagnostics, treatment and epidemiology of Clostridioides (Clostridium) difficile infection in Europe?. J Infect Chemother.

[17] Lessa FC, Mu Y, Bamberg WM, Beldavs ZG, Dumyati GK, Dunn JR, Farley MM, Holzbauer SM, Phipps EC, Wilson LE, Winston LG, Cohen JA, Limbago BM, Fridkin SK, Gerding DN, McDonald LC (2015). Burden of *Clostridium difficile* infection in the United States. N Engl J Med.

[18] Slimings C, Riley TV (2014). Antibiotics and hospital-acquired *Clostridium difficile* infection: update of systematic review and meta-analysis. J Antimicrob Chemother.

[19] Surawicz CM, Brandt LJ, inion DG, Ananthakrishnan AN, Curry SR, Gilligan PH, McFarland LV, Mellow M, Zuckerbraun BS (2013). Guidelines for diagnosis, treatment, and prevention of Clostridium difficile infections. Am J Gastroenterol.

[20] Brown KA, Khanafer N, Daneman N, Fisman DN (2013). Meta-analysis of antibiotics and the risk of community-associated Clostridium difficile infection. Antimicrob Agents Chemother.

[21] Aslam S, Hamill RJ, Musher DM (2005). Treatment of *Clostridium difficile*-associated disease: old therapies and new strategies. Lancet Infect Dis.

[22] Eyre DW, Walker AS, Wyllie D, Dingle KE, Griffiths D, Finney J, O'Connor L, Vaughan A, Crook DW, Wilcox MH, Peto TE (2012). Predictors of first recurrence of *Clostridium difficile* infection: implications for initial management. Infections in Oxfordshire Research Database. Clin Infect Dis.

[23] Johnson S (2009). Recurrent Clostridium difficile infection: a review of risk factors, treatments, and outcomes. J Inf Secur.

[24] Khanna S, Pardi DS, Aronson SL, Kammer PP, Orenstein R, St Sauver JL, Harmsen WS, Zinsmeister AR (2012). The epidemiology of community-acquired *Clostridium difficile* infection: a population-based study. Am J Gastroenterol.

[25] Kelly CP (2012). Can we identify patients at high risk of recurrent *Clostridium difficile* infection?. Clin Microbiol Infect.

[26] Nelson RL, Kelsey P, Leeman H, Meardon N, Patel H, Paul K, Rees R, Taylor B, Wood E, Malakun R. Antibiotic treatment for *Clostridium difficile*-associated diarrhea in adults. Cochrane Database Syst Rev. 2011:CD004610. 10.1002/14651858.CD004610.pub4. 10.1002/14651858.CD004610.pub4 21901692

[27] Louie TJ, Miller MA, Mullane KM, Weiss K, Lentnek A, Golan Y, Gorbach S, Sears P, Shue YK (2011). OPT-80-003 clinical study group. Fidaxomicin versus vancomycin for *Clostridium difficile* infection. N Engl J Med.

